# Multi-level determinants of tuberculosis treatment completion in rural Uganda: A cross-sectional study

**DOI:** 10.1371/journal.pone.0347022

**Published:** 2026-04-20

**Authors:** Munanura Turyasiima, Daniel Muliika, Gaston Turinawe, Miriam Acheng, Antony Ikiriza, Agnes Alinde, Precious Natureeba, Amon Nkwansiibwe, Balbina Gillian Akot, Susan Wendy Wandera Kayizzi, Derrick Asaasira, Shamim Nantege, Iloit Daniel Oode, Hilda Barbara Wesonga, Raymond Kamara Atuhaire, Ronald Kooko

**Affiliations:** 1 Department of Standards Accreditation and Patient Protection, Ministry of Health, Kampala, Uganda; 2 Faculty of Clinical Medicine and Dentistry, Kampala International University, Kampala, Uganda; 3 Faculty of Health Sciences, Uganda Martyrs University, Kampala, Uganda; 4 Division of STD/AIDs Control, Ministry of Health, Kampala, Uganda; 5 Department of Educational Foundations and Psychology, Mbarara University of Science and Technology, Mbarara, Uganda; 6 Department of Health Sciences, Faculty of Science and Technology, Cavendish University Uganda, Kampala, Uganda; 7 Department of Epidemiology, Baylor Foundation, Kampala, Uganda; 8 Department of Epidemiology, Africa CDC, Kampala, Uganda; Kandahar University, Faculty of Medicine, AFGHANISTAN

## Abstract

**Background:**

Tuberculosis (TB) treatment completion rates in high-burden countries like Uganda often fall short of the WHO End TB Strategy target of ≥90%. This study evaluated multilevel determinants of treatment completion to guide evidence-based improvement strategies in rural Western Uganda.

**Methods:**

We conducted a cross-sectional, multi-center analytical study of 224 patients with drug-susceptible TB across four public health facilities in Kakumiro District. Eligible participants had been on treatment for at least six months. Data collected via structured questionnaires were validated against facility TB registers. Multivariable logistic regression identified independent predictors, reported as adjusted odds ratios (AOR) with 95% confidence intervals (CI).

**Results:**

The treatment completion rate was 82.6% (185/224), with a 30.4% TB-HIV co-infection rate. Significant positive predictors included high TB knowledge (AOR = 14.0; 95% CI: 3.06–24.5), high economic status (AOR = 7.2; 95% CI: 1.63–31.5), belief in treatment efficacy (AOR = 6.2; 95% CI: 2.02–18.8), and respectful health worker behavior (AOR = 5.0; 95% CI: 2.15–11.83). Community-level support was critical, specifically religious/community leader advocacy (AOR = 4.2; 95% CI: 1.84–9.51) and community health worker (CHW) home visits (AOR = 3.5; 95% CI: 1.64–7.72). Waiting time less than 30 minutes (AOR = 6.3, 95% CI: 1.91–20.96) also positively impacted TB treatment completion. Major negative predictors were male gender (AOR = 0.3; 95% CI: 0.11–0.86), drug stockouts (AOR = 0.3; 95% CI: 0.11–0.70), belief in traditional cures (AOR = 0.3; 95% CI: 0.13–0.71), and stigma (AOR = 0.4; 95% CI: 0.16–0.80).

**Conclusion:**

Achieving the WHO End TB targets requires integrated, multilevel interventions. Efforts should focus on male-targeted engagement, strengthening supply chains to eliminate drug stockouts, enhancing CHW-led community outreach, and reducing stigma to ensure equitable treatment success.

## Background

Globally, Tuberculosis (TB) remains a leading cause of death from a single infectious agent, surpassing HIV/AIDS, and is among the top ten causes of mortality [1–3]. The burden is disproportionately high in low- and middle-income countries (LMICs), where structural health system weaknesses, socio-economic disparities, and high TB/HIV co-infection rates contribute to ongoing transmission and treatment challenges [1].

While pulmonary TB is the most common form, the infection can also affect other organs such as the brain, kidneys, and spine [1]. Vulnerable populations include individuals living with HIV, those with malnutrition or diabetes, healthcare workers, and people residing in poverty or overcrowded settings that facilitate transmission [3,4]. Beyond its clinical impact, TB imposes profound social and economic burdens, including reduced productivity, catastrophic treatment costs, and persistent stigma [5,6].

Uganda is ranked among the 30 high TB/HIV burden countries worldwide [1]. Despite implementing DOTS (Directly Observed Treatment, Short-course)-based TB control strategies, the country continues to experience treatment completion rates below the World Health Organization (WHO) target of 90% [7,8]. In many districts, high patient volumes, limited resources, and health system gaps contribute to suboptimal completion, increasing the risk of relapse and the emergence of drug-resistant TB [8].

TB treatment completion is shaped by a combination of individual, socio-demographic, community, and health system factors [3,6]. Individual determinants include TB knowledge, beliefs about treatment, use of traditional medicine, treatment side effects, and HIV status [9–11]. Socio-demographic characteristics such as age, gender, education level, income, employment, and place of residence further influence adherence [12–14]. Community factors including community health worker visits, outreach activities, social support, and stigma along with health system factors such as long waiting times, drug stockouts, inadequate counseling, inconsistent follow-up, and poor patient–provider interactions, collectively affect treatment outcomes [15–18].

These barriers remain widespread in Uganda and other developing countries, where socio-economic constraints, persistent stigma, limited community engagement, and systemic health service inefficiencies continue to impede treatment completion [19–21]. Addressing these gaps requires integrated approaches that strengthen health systems while leveraging community-based interventions, patient education, and socio-economic support. Such coordinated measures are essential to achieving the WHO End TB Strategy targets of reducing TB incidence by 80% and TB-related deaths by 90% by 2030 [3,6,22].

Addressing these challenges requires integrated approaches that strengthen health systems while simultaneously leveraging community-based interventions, patient education, and socioeconomic support. Such coordinated measures are essential to achieving the WHO End TB Strategy targets of reducing TB incidence by 80% and TB-related deaths by 90% by 2030 [3,6,22]. This multi-center study investigated the individual, health system, and community factors associated with TB treatment completion in order to inform evidence-based strategies to improve treatment completion, reduce multi-drug-resistant TB (MDR-TB) risk, and accelerate efforts toward national TB control goals and the WHO End TB targets.

## Materials and methods

### Study design and setting

This was a cross-sectional analytical multi-center study between August 2024 to July 2025 conducted in Kakumiro District, located in the Bunyoro sub-region of Midwestern Uganda. The study focused on four high-volume public health facilities: Kakumiro Health Centre IV, Kakindo Health Centre IV, Nalweyo Health Centre III, and Nkooko Health Centre III. These facilities were purposively selected due to their high patient volumes and significant roles in TB diagnosis and treatment in the district. Kakumiro District has an estimated population of 300,000, the majority of whom are subsistence farmers. The district is served by one private-not-for-profit hospital, two Health Centre IVs, fifteen Health Centre IIIs, and nineteen Health Centre IIs.

### Study population and eligibility criteria

The study population comprised patients diagnosed with drug-susceptible tuberculosis (DS-TB) who had been on TB treatment for a minimum of six months at the selected health facilities.

#### Inclusion criteria.

The study included adult patients (aged 18 years and above) diagnosed with drug-susceptible tuberculosis (DS-TB) who had been on treatment for at least six months at selected high-volume health facilities in Kakumiro District. Participants were required to have verifiable medical records and the ability to provide informed consent.

#### Exclusion criteria.

Excluded from the study were individuals with multidrug-resistant (MDR-TB) or extensively drug-resistant TB (XDR-TB), those enrolled in other studies that could affect adherence behavior, patients unable to consent due to cognitive or communication impairments, and individuals who had transferred into the facility from another district after beginning TB treatment elsewhere.

### Sample size determination

Sample size was calculated using the Kish Leslie formula (1965) for cross-sectional studies:


$$n=\frac{Z^{\text{2}}\times P(\text{1}-P)}{d^{\text{2}}}$$

Where:

- *Z* = 1.96 (standard normal deviate at 95% confidence level),
- *P* = 0.904 (treatment completion rate for DS-TB in Kakumiro District, Ministry of Health Information management System (HMIS), January – June 2024),
- *d* = 0.05 (margin of error).

This yielded a minimum sample size of 134. After adjusting for a 10% non-response rate and a design effect of 1.5 due to stratified sampling, the final sample size was 224 participants.

### Sampling procedure

A stratified random sampling approach was used. Eligible patients were stratified by health facility. Within each stratum, participants were selected using simple random sampling, guided by TB treatment registers. Proportional allocation was used to determine the number of participants from each facility. Patients who declined or were ineligible were replaced using re-randomization from the same facility list. This is shown in Table 1.

**Table 1:** Participating Health Facilities.

Health Facility	Sampling Frame (n)	Sample Size (N)
Kakindo HC IV	102	81
Kakumiro HC IV	60	48
Nalweyo HC III	66	52
Nkooko HC III	54	43

### Data collection tools and procedures

Data were collected between August 2024 and July 2025 using a structured, interviewer-administered questionnaire programmed on KoboCollect, a mobile data collection platform. The tool was developed from previous literature, the WHO Advocacy, Communication and Social Mobilization for TB Control Knowledge, Attitude, and Practice survey guide [23,24] and national TB program indicators. The tool was pretested in a neighboring district to ensure validity and contextual appropriateness.

#### Participant approach and recruitment.

Eligible patients were identified through the TB treatment registers of the four participating high-volume health facilities. The list of patients who had completed at least six months of treatment within the last 12 months was extracted in collaboration with the facility TB focal persons.

Patients were contacted at their final clinic visit or during reviews for those who had recently completed treatment. For patients who had already been discharged, contact information recorded in TB registers was used to reach them via community health workers (CHWs), who invited them to participate. Interviews were conducted at the facility or at the participant’s home, depending on convenience and preference.

The purpose of the study was explained to each potential participant in a language they understood (English or Runyoro). Informed consent was obtained prior to data collection. For participants with limited literacy, verbal consent was obtained, documented by the interviewer, and witnessed by an independent third party.

Data collectors were trained health professionals fluent in both English and Runyoro. They received three days of training on study objectives, ethical conduct, interviewing techniques, and use of KoboCollect. To ensure quality, field supervisors conducted daily reviews of submitted forms for completeness and consistency.

#### Operational definition and measurement of treatment completion.

TB treatment completion was assessed according to the World Health Organization (WHO) classification for TB treatment outcomes [25].

*Treatment completed*: A patient who finished the full course of anti-TB drugs without evidence of treatment failure and with or without bacteriological confirmation at the end of treatment.
*Cured*: A patient who completed treatment with bacteriological evidence of cure (smear or culture negative).
*Treatment failed*: A patient whose sputum smear or culture remained or became positive again at month 5 or later during treatment.
*Lost to follow-up (default)*: A patient who interrupted treatment for two consecutive months or more.
*Treatment success*: The sum of “cured” and “treatment completed” patients.

In this study, TB treatment completion was operationalized as patients who self-reported completion of the full prescribed course and whose records in the facility TB register confirmed treatment completion or cure (that is, met WHO “treatment success” criteria). Participants who did not complete or who defaulted were classified as non-completers.

#### Data verification and quality control.

Self-reported responses were cross-checked against facility TB registers to validate treatment completion status. Supervisors conducted random spot-checks and back-checks to ensure data accuracy. Daily data synchronization allowed for timely review and resolution of discrepancies.

### Study variables

The dependent variable was TB treatment completion among patients in high-volume health facilities in Kakumiro District. This was defined as the proportion of patients who completed the full course of TB treatment within the prescribed timeframe. Respondents were asked, “*Have you completed your full TB treatment course*?” (Yes/No). A “Yes” response was verified against the facility TB treatment register. Based on these responses, participants were categorized as either having completed or not completed their TB treatment.

**Fig 1:**
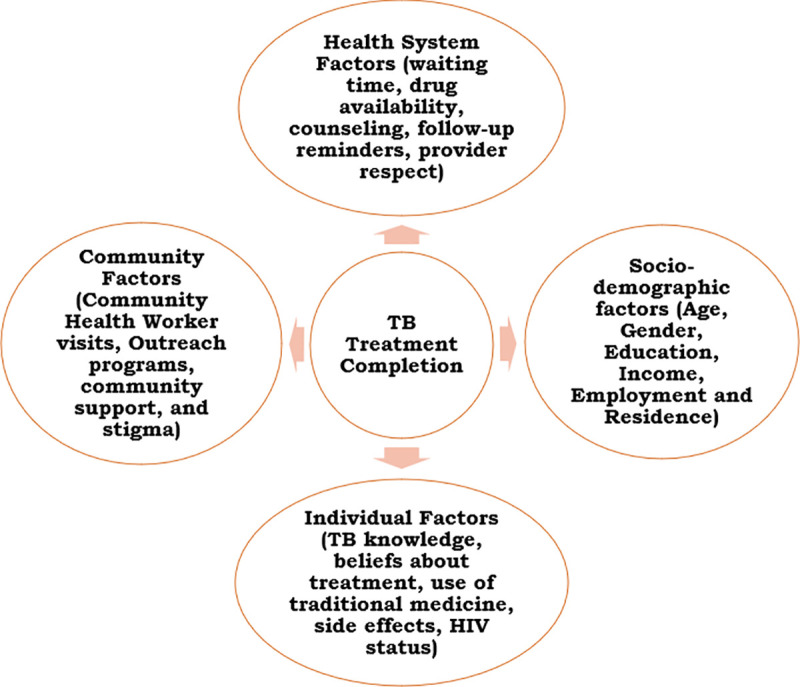
Conceptual Framework of the Determinants of TB Treatment Completion.

The independent variables based on relevant literature search and the WHO End TB strategy 2015 [6] included socio-demographic factors (age, gender, education, income, employment, residence), individual factors (TB knowledge, beliefs about treatment, use of traditional medicine, side effects, HIV status), health system factors (waiting time, drug availability, counseling, follow-up reminders, provider respect), and community factors (community health worker (CHW) visits, outreach programs, community support, and stigma). This is shown in Fig 1.

Economic status was assessed using household consumption expenditure per adult equivalent, benchmarked against the international poverty line of US$3.0 per person per day [26]. Participants were categorized as having low (<US$3.0/day) or high (≥ US$3.0/day) economic status. For context, the median monthly cash and in-kind wage in Uganda is UGX 260,000, approximately equal to the international poverty income line [26,27].

TB knowledge was assessed using an interviewer-administered questionnaire adapted from the WHO Advocacy, Communication and Social Mobilization for TB Control Knowledge, Attitude, and Practice survey guide [23,24]. Knowledge was measured using ten items, each scored as 1 for a correct response and 0 for incorrect or “don’t know” responses, yielding a total score of 0–10. Total scores were converted to proportions and categorized as poor (≤30%), fair (31–60%), or good (>60%) TB knowledge.

### Ethics statement

Ethical approval for this study was obtained from the Mildmay Research Ethics Committee (Reg No: MUREC-2025–805). Administrative clearance was granted by the Kakumiro District Health Office. All participants were informed about the study objectives, procedures, potential risks, and benefits prior to participation. Informed consent was obtained from each participant before data collection. For participants with low literacy, verbal consent was documented by the interviewer and witnessed by an independent third party. Participation was voluntary, and confidentiality was maintained throughout the study. All data were anonymized and securely stored.

### Data management and analysis

Data were analyzed in SPSS version 26 using descriptive, bivariate, and multivariable procedures. Descriptive statistics summarized categorical and continuous variables. Bivariate associations with TB treatment completion were assessed using binary logistic regression, and variables with p < 0.05 were included in the multivariable model. Multicollinearity was checked using Variance Inflation Factors (VIFs), all of which were below 5, indicating no collinearity concerns. A multivariable logistic regression model was then used to identify independent predictors, with Adjusted Odds Ratios (AORs) and 95% confidence intervals (CIs) reported. Model fit was assessed using the Hosmer–Lemeshow goodness-of-fit test, which indicated adequate calibration of the final model (*χ² = 6.21, p = 0.623*), and results were presented in tables. Records with missing data for variables of interest were excluded from the analysis (complete-case analysis).

### Study limitations

The study relied partly on self-reported data, which may be subject to recall or social desirability bias. These were mitigated by triangulating responses with facility records and using memory aids. Additionally, the exclusion of MDR-TB patients may limit generalizability to all TB cases.

## Results

### Socio-demographic characteristics of respondents

A total of 224 respondents participated in the study. The mean age was 43.4 years (±15.7), with a median of 42 years (IQR: 30–55). The majority were female (54.5%) and married (64.3%). Most participants were from Kakindo Health Centre IV (36.2%) and identified as Catholic (55.8%). Regarding education, 49.6% had attained primary education, while 26.8% had no formal education. Most respondents resided in rural areas (86.6%), reported low economic status (71.0%), and were not formally employed (77.7%). Notably, 30.4% were living with HIV. This is shown in Table 2.

**Table 2:** Socio-demographic characteristics of the respondents (N = 224).

Variables	Frequency (n)	Percentage (%)
Age		
Age (Mean ± SD)	43.4 ± 15.7	
Age (Median, IQR)	42 (30,5-55)	
18-37	87	38.8
38-57	95	42.4
≥58	42	18.8
Gender		
Female	122	54.5
Male	102	45.5
Marital status		
Single	44	19.6
Married	144	64.3
Divorced	23	10.3
Widowed	13	5.8
Health facility		
Kakindo	81	36.2
Kakumiro	48	21.4
Nalweyo	52	23.2
Nkooko	43	19.2
Religion		
Anglican	44	19.6
Catholic	125	55.8
Muslim	10	4.5
Pentecostal	35	15.6
SDA	10	4.5
Level of education		
No formal education	60	26.8
Primary	111	49.6
Secondary	30	13.4
Tertiary	23	10.3
Area of residence		
Rural	194	86.6
Semi-urban	30	13.4
Economic status		
Low	159	71.0
High	65	29.0
Employment status		
Formal employment	50	22.3
No formal employment	174	77.7
People living with HIV		
No	156	69.6
Yes	68	30.4

### TB treatment completion rate

**Fig 2:**
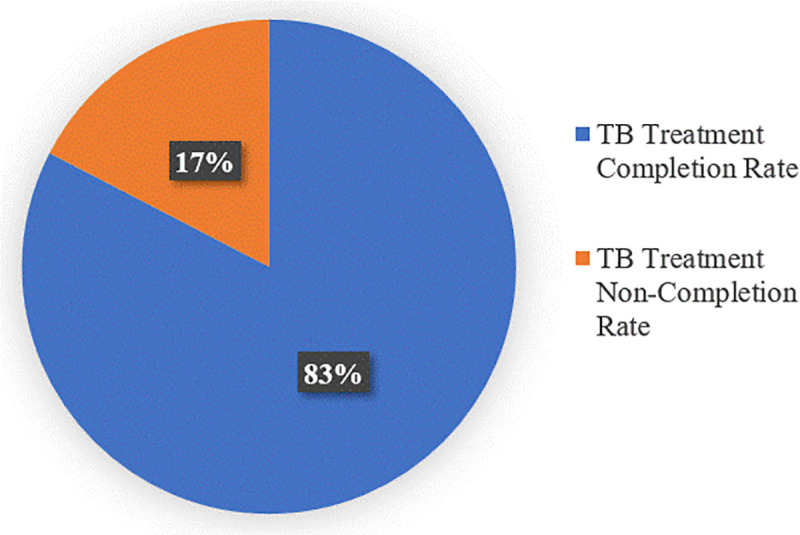
Prevalence of TB treatment completion.

Among the 224 TB patients surveyed, 185 (82.6%) completed their full course of treatment, while 39 (17.4%) did not. This completion rate, though relatively high, remains below the WHO target of 90%. This is shown in Fig 2.

### Factors associated with TB treatment completion

Descriptive, bivariate, and multivariable analyses were conducted to identify factors associated with TB treatment completion. Binary logistic regression was used to examine associations between treatment completion and individual, health system, and community factors. Variables significant at *p* < 0.05 in bivariate analysis were included in the multivariable model, with adjusted odds ratios (AORs) and 95% confidence intervals (CIs) reported to determine independent predictors.

Multivariable logistic regression identified several socio-demographic, individual, health system, and community factors significantly associated with TB treatment completion. Among socio-demographic factors, male patients were less likely to complete treatment than females (AOR = 0.3, 95% CI: 0.11–0.86, p = 0.025), while those with higher economic status were more likely to complete treatment compared to those with lower status (AOR = 7.2, 95% CI: 1.63–31.52, p = 0.009). At the individual level, patients with greater TB knowledge had substantially higher odds of completing treatment (AOR = 14.0, 95% CI: 3.06–24.50, p = 0.001), and belief in the effectiveness of TB treatment was also a strong positive determinant (AOR = 6.2, 95% CI: 2.02–18.78, p = 0.001). Conversely, belief that TB could be cured by traditional medicine was associated with lower odds of completion (AOR = 0.3, 95% CI: 0.13–0.71, p = 0.006). Experiencing side effects and discontinuing medication after symptom relief were negatively associated with completion but did not remain significant in the final model. This is shown in Table 3.

**Table 3:** Respondents’ Socio-demographic and individual health factors associated with TB treatment completion rates.

Variable	TB treatment completion rates		Crude OR (95% CI)	P-value	Adjusted OR (95%CI)	P-value
	Completion N= 185 (%)	Non-completion N= 39 (%)				
**Socio-demographic factors**						
Level of education						
No formal education	45 (75.0)	15 (25.0)	Reference	-	-	-
Primary	93 (83.8)	18 (16.2)	1.7 (0.80-3.73)	0.168	-	-
Secondary	26 (86.7)	4 (13.3)	2.2 (0.65-7.22)	0.208	-	-
Tertiary	21 (91.3)	2 (8.7)	3.5 (0.73-16.72)	0.116	-	-
Area of residence						
Rural	159 (82.0)	35 (18.0)	Reference	-	-	-
Semi-urban	26 (86.7)	4 (13.3)	1.4 (0.47-4.36)	0.529	-	-
Economic status						
Low (household consumption expenditure < US$3.0/day)	124 (78.0)	35 (22.0)	Reference	-	Reference	-
High (household consumption expenditure > US$3.0/day)	61 (93.8)	4 (6.2)	4.3 (1.46-12.6)	0.008	7.2 (1.63-31.5)	0.009
No. of dependents						
≤3	67 (81.7)	15 (18.3)	Reference	-	-	-
4-6	99 (82.5)	21 (17.5)	1.1 (0.51-2.19)	0.885	-	-
≥7	19 (86.4)	3 (13.6)	1.4 (0.37-5.42)	0.610	-	-
Employment status						
Formal employment	41 (82.0)	9 (18.0)	Reference	-	-	-
Non-Formal employment	144 (82.8)	30 (17.2)	0.9 (0.42-2.16)	0.901	-	-
**Individual related health factors**						
Currently living with HIV						
No	127 (81.4)	29 (18.6)	Reference	-	-	-
Yes	58 (85.3)	10 (14.7)	1.3 (0.61-2.90)	0.482	-	-
Alcohol consumption						
No	90 (90.9)	9 (9.1)	Reference	-	Reference	-
Yes	95 (76.0)	30 (24.0)	0.3 (0.14-0.70)	0.005	0.5 (0.17-1.36)	0.169
Knowledge about TB treatment						
Poor	31 (60.8)	20 (39.2)	Reference	-	Reference	-
Fair	88 (85.4)	15 (14.6)	3.8 (1.73-8.30)	0.001	3.4 (1.14-9.94)	0.029
Good	66 (94.3)	4 (5.7)	10.6 (3.35-33.80)	<0.001	14.0 (3.06-24.50)	0.001
Smokes cigarette						
No	145 (84.3)	27 (15.7)	Reference	-	-	-
Yes	40 (76.9)	12 (23.1)	0.6 (0.29-1.33)	0.222	-	-
Understand importance of TB treatment completion						
	27 (60.0)	18 (40.0)	Reference	-	Reference	-
Yes	158 (88.3)	21 (11.7)	5.0 (2.37-10.62)	<0.001	2.1 (0.68-6.50)	0.195
Experiences side effects from the TB medication						
No	110 (87.3)	16 (12.7)	Reference	-	Reference	-
Yes	75 (76.5)	23 (23.5)	0.5 (0.24-0.96)	0.037	0.7 (0.26-1.91)	0.488
Believe TB treatment is effective						
No	14 (41.2)	20 (58.8)	Reference	-	Reference	-
Yes	171 (90.0)	19 (10.0)	12.9 (5.60-29.53)	<0.001	6.2 (2.02-18.78)	0.001
Treatment duration too long						
No	137 (89.5)	16 (10.5)	Reference	-	Reference	-
Yes	48 (67.6)	23 (32.4)	0.2 (0.12-0.50)	<0.001	0.4 (0.14-1.19)	0.099
Stopped medication after feeling better						
No	162 (88.0)	22 (12.0)	Reference	-	Reference	-
Yes	23 (57.5)	17 (42.5)	0.2 (0.09-0.40)	<0.001	0.4 (0.11-1.28)	0.117
Aware about risks of stopping medication						
No	43 (67.2)	21 (32.8)	Reference	-	Reference	-
Yes	142 (88.8)	18 (11.3)	3.9 (1.88-7.88)	<0.001	1.6 (0.56-4.46)	0.390
Believe TB can be cured by traditional medicine						
No	147 (89.6)	17 (10.4)	Reference	-	Reference	-
Yes	38 (63.3)	22 (36.7)	0.2 (0.10-0.41)	<0.001	0.3 (0.13-0.71)	0.006
Felt stigmatized for having TB						
No	134 (89.3)	16 (10.7)	Reference	-	Reference	-
Yes	51 (68.9)	23 (31.1)	0.3 (0.13-0.54)	<0.001	0.6 (0.25-1.43)	0.250

Key: (-) Indicates that the p-value or adjusted odds ratio (AOR) is not applicable because the variable was used as a reference category or was not statistically significant at bivariate analysis (crude odds ratio, p > 0.05).

Key health system factors associated with treatment completion included waiting time, counseling, and drug availability. Patients who waited less than 30 minutes at the facility were more likely to complete treatment (AOR = 6.3, 95% CI: 1.91–20.96, p = 0.003), as were those who received follow-up reminders (AOR = 2.7, 95% CI: 1.13–6.67, p = 0.026). Regular drug supply and access to counseling services also enhanced treatment completion (AOR = 4.3, 95% CI: 1.74–10.61, p = 0.002), while drug stockouts significantly reduced the likelihood of completion (AOR = 0.3, 95% CI: 0.11–0.70, p = 0.006). Respectful behavior from health workers further strengthened adherence (AOR = 5.0, 95% CI: 2.15–11.83, p < 0.001).

Community-related factors also played an important role. Participation in TB outreach and sensitization activities was positively associated with completion (AOR = 3.6, 95% CI: 1.27–6.72, p = 0.001), as was receiving home visits from community health workers (AOR = 3.5, 95% CI: 1.64–7.72, p = 0.001) and support from community or religious leaders (AOR = 4.2, 95% CI: 1.84–9.51, p = 0.001). In contrast, patients who experienced community stigma were less likely to complete treatment (AOR = 0.4, 95% CI: 0.16–0.80, p = 0.012). This is shown in Table 4.

**Table 4:** Health system and community health related factors associated with TB treatment completion rates.

Variable	TB treatment completion rates		Crude OR (95% CI)	P-value	Adjusted OR (95%CI)	P-value
	Completion N= 185 (%)	Non-completion N= 39 (%)				
**Health system related factors**						
Easy access to treatment center						
No	49 (77.8)	14 (22.2)	Reference	-	-	-
Yes	136 (84.5)	25 (15.5)	1.6 (0.75-3.23)	0.237	-	-
Distance to treatment center						
More than 5 km	76 (74.5)	26 (25.5)	Reference	-	Reference	-
1-5 km	67 (88.2)	9 (11.8)	2.5 (1,12-5.82)	0.027	2.9 (1.10-7.65)	0.052
Less than 1 km	42 (91.3)	4 (8.7)	3.6 (1.17-10.00	0.025	3.1 (0.85-11.38)	0.085
Health worker respectfulness						
No	35 (61.4)	22 (38.6)	Reference	-	Reference	-
Yes	150 (89.8)	17 (10.2)	5.5 (2.67-11.53)	<0.001	5.0 (2.15-11.83)	<0.001
Health worker provided Information on treatment schedule						
No	12 (66.7)	6 (33.3)	Reference	-	-	-
Yes	173 (84.0)	33 (16.0)	2.6 (0.92-7.48)	0.072	-	-
Waiting time at treatment center						
More than 1 hour	21 (60.0)	14 (40.0)	Reference	-	Reference	-
30 minutes to 1 hour	82 (82.0)	18 (18.0)	3.0 (1.30-7.09)	0.010	2.4 (0.87-6.42)	0.090
Less than 30 minutes	82 (92.1)	7 (7.9)	7.8 (2.80-21.79)	<0.001	6.3 (1.91-20.96)	0.003
Adequate side effect information						
No	29 (78.4)	8 (21.6)	Reference	-	-	-
Yes	156 (83.4)	31 (16.6)	1.4 (0.58-3.32)	0.461	-	-
Follow-up reminder						
No	41 (63.1)	24 (36.9)	Reference	-	Reference	-
Yes	144 (90.6)	15 (9.4)	5.6 (2.70-11.69)	<0.001	2.7 (1.13-6.67)	0.026
Medicine shortages						
No	157 (90.8)	16 (9.2)	Reference	-	Reference	-
Yes	28 (54.9)	23 (45.1)	0.1 (0.06-0.26)	<0.001	0.3 (0.11-0.70)	0.006
Offer counseling services as part of the treatment						
No	39 (59.1)	27 (40.9)	Reference	-	Reference	-
Yes	146 (92.4)	12 (7.6)	8.4 (3.91-18.13)	<0.001	4.3 (1.74-10.61)	0.002
Attitude of Health workers						
Not friendly	19 (59.4)	13 (40.6)	Reference	-	Reference	-
Friendly	83 (82.2)	18 (17.8)	3.2 (1.32-7.5)	0.010	1.6 (0.52-5.06)	0.410
Very friendly	83 (91.2)	8 (8.8)	7.1 (2.58-19.5)	<0.001	1.7 (0.48-6.56)	0.385
Health worker explains the benefits of completing TB treatment						
No	38 (64.4)	21 (35.6)	Reference	-	Reference	-
Yes	147 (89.1)	18 (10.9)	4.5 (2.19-9.31)	<0.001	1.6 (0.64-4.16)	0.309
Comfortable discussing concerns with health workers						
No	31 (70.5)	13 (29.5)	Reference	-	Reference	-
Yes	154 (85.6)	26 (14.4)	2.5 (1.15-5.36)	0.020	1.5 (0.54-4.24)	0.429
**Community related factors**						
Family encouragement						
No	32 (91.4)	3 (8.6)	Reference	-	Reference	-
Yes	153 (81.0)	36 (19.0)	1.4 (1.02-1.91)	0.037	0.3 (0.09-1.29)	0.113
Community outreach/ awareness programs about TB treatment						
No	52 (69.3)	23 (30.7)	Reference	-	Reference	-
Yes	133 (89.3)	16 (10.7)	3.7 (1.80-7.51)	<0.001	3.6 (1.27-6.72)	0.001
Access to support groups						
No	127 (83.0)	26 (17.0)	Reference	-	-	-
Yes	58 (81.7)	13 (18.3)	0.9 (0.44-1.90)	0.809	-	-
Community health worker home visit during treatment						
No	59 (69.3)	26 (30.6)	Reference	-	Reference	-
Yes	126 (90.6)	13 (9.4)	4.3 (2.05-8.90)	<0.001	3.5 (1.64-7.72)	0.001
Community/Religious leaders’ support towards TB treatment						
No	41 (62.1)	25 (37.9)	Reference	-	Reference	-
Yes	144 (91.1)	14 (8.9)	6.3 (2.99-13.15)	<0.001	4.2 (1.84-9.51)	0.001
Stigmatized by community for having TB						
No	171 (84.7)	31 (15.3)	Reference	-	Reference	-
Yes	14 (63.6)	8 (36.4)	0.3 (0.12-0.82)	0.018	0.4 (0.16-0.80)	0.012
Social pressure not to complete TB treatment						
No	151 (84.4)	28 (15.6)	Reference	-	Reference	-
Yes	34 (75.6)	11 (24.4)	1.8 (1.20-2.62)	0.004	0.4 (0.18-1.03)	0.060
Treatment information/awareness message from Radio/TV						
No	56 (68.3)	26 (31.7)	Reference	-	Reference	-
Yes	129 (90.8)	13 (9.2)	4.6 (2.21-9.62)	<0.001	2.0 (0.84-4.76)	0.115
Cultural/Religious practices discouraging TB treatment						
No	178 (83.2)	36 (16.8)	Reference	-	-	-
Yes	7 (70.0)	3 (30.0)	0.5 (0.12-1.91)	0.293	-	-

Key: (-) Indicates that the p-value or adjusted odds ratio (AOR) is not applicable because the variable was used as a reference category or was not statistically significant at bivariate analysis (crude odds ratio, p > 0.05).

## Discussion

This multi-center study assessed the prevalence of tuberculosis (TB) treatment completion and examined individual, health system, and community-level factors associated with it.

### Overall TB treatment completion and burden of HIV–TB co-infection

The TB treatment completion rate in this cohort was 82.6%, which remains below both the national target (≥ 85%) and the global End TB Strategy success standard of ≥ 90% [3,7]. Nonetheless, this estimate aligns with the documented sub‑national variability in Uganda, where previous studies have reported treatment success or completion rates in the range of 66.7% to 89.8%, underscoring persistent heterogeneity across regions [28,29].The prevalence of HIV–TB co‑infection in this study was 30.4%, which, while somewhat lower than Uganda’s earlier national estimate of 40%, remains very high in comparison to global figures. For example, WHO data indicate that only 8.2% of incident TB cases worldwide are among people living with HIV [8,30]. This discrepancy stresses the disproportionate burden of HIV/TB co‑infection in Uganda, particularly in resource-limited settings.

### Socio-demographic determinants of TB treatment completion

Gender and economic status were the dominant socio‑demographic determinants of TB treatment completion. Being male was negatively associated with TB treatment completion, consistent with many studies that men are disadvantaged in seeking and/or accessing TB care in many settings [12]. Male disadvantage in completion mirrors broader sex‑differentiated care‑seeking patterns driven by occupational mobility, sociocultural expectations, and stigma‑related delays [12,13,31]. Patients of higher socioeconomic status were 7.2 times more likely to complete TB treatment than those of lower status. This aligns with findings from eastern Uganda, where individuals in the highest wealth quintile had a 58% lower risk of unsuccessful treatment [32]. Economic resilience likely enhances adherence by reducing indirect costs such as transport and lost income, while supporting better nutrition and psychosocial stability [14,33,34]. The attenuation of other demographic factors after adjustment further suggests that structural economic barriers and gendered differences in access may outweigh educational or residential influences in similar rural contexts.

### Individual knowledge, beliefs, and cognitive factors influencing TB treatment completion

At the individual level, patients with high knowledge of TB treatment were significantly 14 times more likely to complete therapy compared with those with low knowledge. Similarly, individuals who believed in the efficacy of TB treatment had markedly higher odds of completion. These cognitive and perceptual factors strongly predicted treatment completion, consistent with health-literacy pathways documented elsewhere [9,10,35,36] and aligned with the Health Belief Model’s construct of perceived benefit [37]. Conversely, attribution of cure to traditional medicine significantly reduced the likelihood of treatment completion by 70%, echoing findings from South Africa, Ghana, and other settings where traditional healing beliefs influenced TB care-seeking and adherence patterns [11,38,39]. Rather than framing traditional practices purely as barriers, evidence suggests that constructive engagement of traditional healers in education and referral may convert a competing pathway into a complementary one [15,38].

### Health system performance and quality of care factors

Health system performance factors such as waiting time, follow-up reminders, drug availability, counseling, and respectful provider interactions showed strong and quantifiable associations with TB treatment completion. Patients who experienced waiting times under 30 minutes had 6.3 times higher odds of completing treatment, consistent with studies from Nigeria and other low-resource settings where reduced waiting times improved adherence [18,40]. Shorter waiting times typically reflect greater service efficiency and patient-centered flow [41,42]. Similarly, individuals who received follow-up reminders via SMS or phone calls were 2.7 times more likely to complete therapy, aligning with evidence that digital and community health worker (CHW)-supported reminder systems enhance TB treatment completion [43,44]. Such reminders operationalize WHO’s patient-support package, leveraging low-cost digital and community mechanisms to mitigate forgetfulness, competing priorities, and early symptom-driven discontinuation [3,44,45].

In addition, access to counseling services increased the odds of completion more than fourfold, consistent with findings from similar contexts where structured counseling improves adherence [46]. Respectful and supportive behavior from health workers was also highly predictive, with exposed patients having five times greater odds of completing treatment. Counseling and empathetic provider communication help address fears regarding treatment duration and side effects, strengthening the therapeutic alliance – an adherence determinant widely documented in qualitative and mixed-methods studies [36,47–49]. Conversely, drug stockouts substantially undermined adherence, reducing the odds of completion by 70%, highlighting the importance of uninterrupted drug availability in sustaining therapeutic momentum and reinforcing patient trust [10,15]. Collectively, these modifiable system levers align with global quality-of-care frameworks that emphasize timeliness, continuity, reliability, and dignity in service delivery.

### Community-level influences on TB treatment completion

Community-related factors also significantly influenced TB treatment completion. Participation in TB outreach and sensitization activities was associated with 3.6 times higher odds of completing treatment, while receiving home visits from community health workers similarly increased the likelihood of completion by 3.5 times. These findings are supported by evidence from other settings, which highlight the critical role of community engagement as an active component in the implementation of interventions for TB prevention, care, and adherence [46]. In addition, support from religious leaders significantly increased the likelihood of completing TB treatment, with patients receiving such backing having 4.2 times greater odds of treatment completion. Conversely, experiencing community stigma markedly reduced adherence, with affected patients showing 60% lower odds of completing therapy, a factor consistent with findings from other studies [17,21,50].

Strengthening community engagement through frequent outreach, culturally tailored messaging, and active involvement of community, religious, and traditional leaders has been shown to reduce stigma and promote TB treatment completion, partly by normalizing survivor experiences [15–17]. Targeted CHW home visits especially for patients with early missed doses, socioeconomic challenges, or reliance on traditional remedies further bolster adherence [9,51]. Additionally, there is evidence in comparable settings that scaling interoperable digital adherence tools, monitoring stigma indicators, and integrating adherence metrics into routine performance dashboards have contributed to sustained improvements in TB treatment outcomes [1,3].

## Strengths and limitations

Strengths include focus on high‑volume facilities (enhancing programmatic relevance), multi‑level variable capture (patient, system, community), and verification of self‑reported completion against registers, mitigating misclassification. Limitations inherent to the cross‑sectional design preclude temporal inference; some associations may reflect reverse causality (for example, counseling exposure could both promote and be triggered by adherence concerns). Potential residual confounding (nutritional status, mental health, transport cost granularity) may persist. Findings are most generalizable to rural, high‑burden districts with similar service organization; extrapolation to urban or MDR‑TB populations should be cautiousClick or tap here to enter text.. Social desirability bias in self‑reported beliefs and stigma experiences cannot be fully excluded despite validation measures.

### Programmatic and global relevance

The convergence of modifiable factors across domains underscores that incremental, siloed interventions (for example education alone) are unlikely to achieve End TB adherence benchmarks. Integrated packages combining rapid service flow redesign, digital or CHW reminder systems, continuous drug supply, structured counseling, male‑tailored engagement, economic risk mitigation, stigma reduction, and strategic inclusion of community and traditional influencers aligned with international recommendations may yield synergistic improvements.

## Recommendations and conclusion

To improve TB treatment completion in rural Uganda, an integrated, multilevel adherence-support package is recommended. At the patient level, continuous, culturally appropriate education should reinforce treatment efficacy, duration rationale, and side-effect management, while addressing misconceptions about traditional cures. Reminder systems via SMS or community health workers should be institutionalized, with targeted engagement strategies for men and economically vulnerable patients.

Health system improvements should prioritize reducing waiting times, ensuring uninterrupted drug supply, and embedding structured counseling into routine care. Strengthening follow-up mechanisms and promoting respectful, patient-centered communication among health workers are essential to sustaining adherence.

At the community level, expanding TB outreach, intensifying CHW home visits, and engaging community and religious leaders as adherence and stigma-reduction champions are critical. Constructive collaboration with traditional healers can also help mitigate competing treatment pathways.

In conclusion, TB treatment completion in Kakumiro District remains below national and global targets, reflecting interconnected patient, health system, and community barriers. Targeted, integrated interventions combining efficient service delivery, reliable drug supply, counseling, digital and community health worker support, stigma reduction, and socioeconomic assistance offer the greatest potential to improve adherence. These findings are likely applicable to other rural and resource-limited settings facing similar challenges, providing evidence to inform strategies that advance TB control, reduce treatment failure, and curb the emergence of drug-resistant disease in comparable high-burden contexts.

## Supporting information

S1 File TB Treatment Completion in Kakumiro District, Uganda. (XLSX)
